# Multiple Reflections for Classical Particles Moving under the Influence of a Time-Dependent Potential Well

**DOI:** 10.3390/e24101427

**Published:** 2022-10-07

**Authors:** Flávio Heleno Graciano, Diogo Ricardo da Costa, Edson D. Leonel, Juliano A. de Oliveira

**Affiliations:** 1 Departamento de Física, Universidade Estadual Paulista (UNESP), Instituto de Geociências e Ciências Exatas, Câmpus de Rio Claro, Av. 24A, 1515, São Paulo 13506-900, SP, Brazil; 2 Instituto Federal do Sul de Minas Gerais (IFSULDEMINAS), Campus Pouso Alegre, Avenida Maria da Conceição Santos nº 900, Bairro Parque Real, Pouso Alegre 37560-260, MG, Brazil; 3 Departamento de Física, Universidade Federal do Paraná (UFPR), Curitiba 80060-000, PR, Brazil; 4 Instituto de Matemática e Estatística da Universidade de São Paulo (IME-USP), Rua do Matão, 1010, São Paulo 05508-090, SP, Brazil; 5 Câmpus de São João da Boa Vista, Universidade Estadual Paulista, Av. Profa. Isette Corrêa Fontão, 505, São Paulo 13876-750, SP, Brazil

**Keywords:** time-dependent potential well, dispersion of the initial conditions, multiple reflections

## Abstract

We study the dynamics of classical particles confined in a time-dependent potential well. The dynamics of each particle is described by a two-dimensional nonlinear discrete mapping for the variables energy $e_{n}$ and phase $\phi_{n}$ of the periodic moving well. We obtain the phase space and show that it contains periodic islands, chaotic sea, and invariant spanning curves. We find the elliptic and hyperbolic fixed points and discuss a numerical method to obtain them. We study the dispersion of the initial conditions after a single iteration. This study allows finding regions where multiple reflections occur. Multiple reflections happen when a particle does not have enough energy to exit the potential well and is trapped inside it, suffering several reflections until it has enough energy to exit. We also show deformations in regions with multiple reflection, but the area remains constant when we change the control parameter $N_{C}$. Finally, we show some structures that appear in the $e_{0}e_{1}$ plane by using density plots.

## 1. Introduction

In the area of chaos and dynamical systems [1], we have several models that deal with multiple reflections. The Fermi–Ulam model is one of these models [2,3,4]; it corresponds to a classical particle with mass *m*, confined between two rigid walls, where one of them stays at a fixed position while the other moves accordingly to a cosine function. In this system, the particle can hit several times the moving wall before leaving the reflections zone. Another example is the bouncer model [5], which is an alternative model to the Fermi–Ulam model. Here, we consider a particle of mass *m* moving vertically under the influence of a gravitational field *g*. In this movement, the particle collides with a horizontal surface oscillating vertically in an interval that defines the reflections zone. When a particle has more than one reflections with this oscillating surface before leaving the reflections zone, we say that the particle has suffered multiple reflections.

We can also consider billiard systems [6], which describes the movement of a particle inside a closed region, where this particle collides with the billiard boundary (which can have circular, elliptical, or oval shapes) [7]. In these systems, we can also observe the occurrence of multiple reflections, and it happens when we apply a time-dependent perturbation in the boundaries [8,9,10].

Multiple reflections can also be observed when studying classical particles confined within time-dependent potential well or barriers. The dynamics of these particles is an object of study for many researchers [11,12,13,14,15,16]. We can find applications in classical mechanics, quantum mechanics and electromagnetism [17,18,19]. In Ref. [19], the authors show classical particles interacting with one, two, or an infinite chain of squared potential wells, where the bottoms oscillate periodically in time. The dynamics of these systems are described by a two-dimensional mapping [20,21] that generates a phase space of mixed-type, e.g., containing chaotic sea, stability islands, and invariant spanning curves.

In these systems, it is possible to study the survival probability of an ensemble of particles [16]. Through these studies, an outstanding result was obtained, showing that the histograms of escaping particles are scaling invariant [22,23]. We consider that a particle escaped if its energy is higher than a given value. These histograms grow fast until they reach their maximum value and then decrease to zero for sufficiently long times. It is also possible to obtain other scaling properties, for example, when studying the deviation of the average energy [24]. Scaling exponents were also found: namely, acceleration, saturation, and crossover exponents. After an appropriate rescaling of the axis, a collapse of all curves in a single universal plot was possible [24], showing a scaling invariance. The authors in Ref. [25] showed, for a time-dependent potential barrier, that there are some regions in the phase space presenting multiple reflections. Inspired by this work, in this paper, we study a potential well. We divide this system into two regions; in the first region, the bottom has an oscillatory behavior. The second region has a constant potential energy.

In this paper, we intend to answer the following questions: What are the initial conditions in the time-dependent potential well that lead to multiple reflections? Are there preferred regions where these multiple reflections occur? We also show that the multiple reflections are essential to understand the density plots in the $e_{0}e_{1}$ plane. Finally, we study the histogram for the number of multiple reflections, which present a power-law behavior as a function of *i*, which is the integer that satisfies the reflection condition. We will show that these histograms are scaling invariant, e.g., they exhibit a universal behavior for any combination of parameters.

Our work has the following organization: We present the model in Section 2, and we describe, in detail, how to construct the mapping. After that, some results are drawn in Section 3. Section 4 shows our final remarks.

## 2. The Model and Mapping

**Figure 1:**
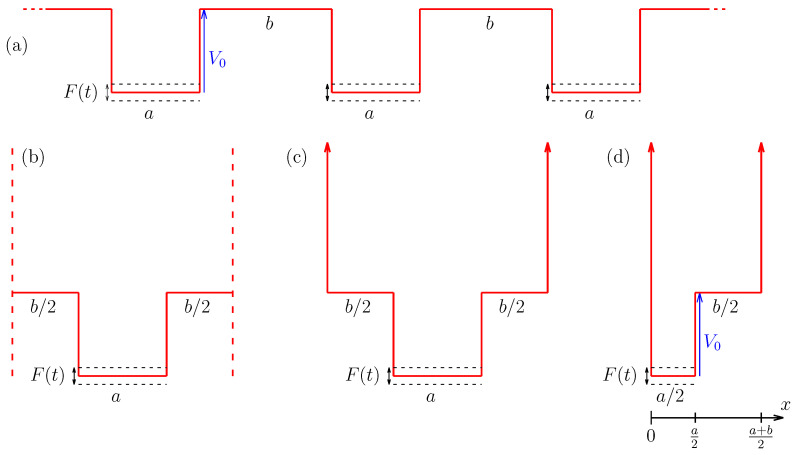
(**a**) A sequence containing an infinite number of time-dependent potential wells, where *b* is the distance between potential wells, *a* is the width of the moving wall, $V_{0}$ is the depth of the potential well, and the bottom of the moving well moves accordingly $F\left(t\right)=V_{1}\cos\left(\omega t\right)$. (**b**) After observing some symmetries, one can take a unique time-dependent potential well with an infinity potential on both sides. In (**c**), we use another symmetry, where we split the potential well in the middle. In (**d**), we have the time-dependent potential well obtained by a cut in the center of (**c**) where we consider an infinite potential. This last time-dependent potential well is our object of study, which is our object of study.

We consider a classical particle moving under the influence of a potential energy V(x,t). Figure 1a considers a chain of infinity time-dependent potential wells, where the bottoms are synchronously moving according to $F\left(t\right)=V_{1}\cos\left(\omega t\right)$. According to Figure 1a, we consider $V_{0}$ as the height of the potential well. Observe that the particle can move freely along the x-axis, and the dynamics leads to diffusion in space. We emphasize that the different kinds of potential shapes lead to similar dynamics in energy and time plane. One can also assume a single oscillating square well with periodic boundary conditions, as shown in Figure 1b, with *a* and *b* giving the widths of the potential well. In this new figure, we can imagine a particle moving from left to right. If this particle reaches the dashed vertical line in the right, it is instantly moved to the left dashed line, and the particle continues going from left to right, analogously to periodic boundary conditions. Note that it is analogous to the case shown in Figure 1a. With this in mind, one can change the boundary conditions shown in Figure 1b, where we can consider an infinite potential as boundaries. As an example, we are going to admit a particle that travels from left to right. If it reaches the infinite potential on the right-hand side, it experiences an elastic reflection, that is: it starts to move in the opposite direction after the shock without losing energy, changing its direction and traveling backward (from right to left) [19].

The potential well shown in Figure 1c presents an important symmetry. Observe that we can split it into two halves, where one of these halves is shown in Figure 1d. We consider an infinite potential in x=0. The dynamics shown in Figure 1d is exactly the same as shown in Figure 1a–c in energy and time plane. In this paper, we are going to consider Figure 1d, where the potential energy of the particle is given by

(1) $$V\left(x,t\right)=\left\{\begin{array}{c} \infty ,\;\mathrm{if}\;x\leq 0\;\mathrm{or}\;x\geq (a+b)/2\\ F(t),\;\mathrm{if}\;0<x<a/2\\ V_{0},\;\mathrm{if}\;a/2\leq x<\left(a+b\right)/2 \end{array}\right..$$

*a*, *b*, ω, $V_{1}$ and $V_{0}$ are control parameters. The most frequently potential well model studied is the model of Figure 1c [12]. However, we will see later that using the model of Figure 1d and fixing the Poincaré section on the position $\frac{a+b}{2}$ make the results the same, but the fixed points are symmetrical, facilitating our studies.

We consider a classical particle starting at x=(a+b)/2, with initial energy $E_{n}>V_{0}$ and an initial time $t=t_{n}$. Each time the particle reaches the position x=(a+b)/2, we save the energy *E* and time *t*; then, this position will be our Poincaré section [26,27], which is different from previous publications [25]. This change is necessary because some observables in this paper are better observed when using this specific Poincaré section. With all these observations in mind, we start by showing how to obtain the new mapping.

Assuming the particle starts at x=(a+b)/2, it travels to the left with initial velocity $v_{0}=\sqrt{2K_{0}/m}$, where $K_{0}=E_{n}-V_{0}$ is the initial kinetic energy. We name $\Delta t_{b}=b/\left(2v_{0}\right)$ as the time to travel the distance b/2 with velocity $V_{0}$.

The particle reaches the position x=a/2, where it suffers an abrupt change in the potential energy after entering the moving well. Here, it is necessary to consider the conservation of mechanical energy, which leads to

(2) $$K_{a}=E_{n}-V_{1}\cos\left[\omega \left(t_{n}+\Delta t_{b}\right)\right],$$

which is the kinetic energy of the particle when entering the moving well. After that, the particle travels the distance a/2 with constant velocity $v_{a}=\sqrt{2K_{a}/m}$ (no forces are acting in the system), reaches the infinity potential at x=0 and goes to the right, traveling again a distance a/2. The particle is back to the position x=a/2, with mechanical energy given by

(3) $$E_{a}=K_{a}+V_{1}\cos\left[\omega \left(t_{n}+\Delta t_{b}+\Delta t_{a}\right)\right].$$

where $\Delta t_{a}$ is the time taken for the particle to go from x=a/2 to x=0 and back to x=a/2. If the energy is not enough to trespass the potential energy $V_{0}$, i.e., $E_{a}\leq V_{0}$, then the particle is deflected and goes to the left. The particle stays trapped until its energy at x=a/2 is higher than $V_{0}$, where it can be expressed by solving

(4) $$E_{a}=K_{a}+V_{1}\cos\left[\omega \left(t_{n}+\Delta t_{b}+i\Delta t_{a}\right)\right]>V_{0},$$

where *i* is the smallest integer number that satisfies this condition. Therefore, the particle is trapped inside the moving well until it has enough energy to escape. Taking this into account, we define the number of multiple reflections as

(5) $$n_{suc}=i-1.$$

If i=1 (or $n_{suc}$ = 0), we do not have multiple reflections, i.e., the particle leaves the reflections zone directly. If i=2, the particle undergoes multiple reflections before escaping the moving well. If i=3, the particle undergoes two multiple reflections before escaping, and so on.

When the particle has enough energy to trespass the potential energy $V_{0}$, we observe an abrupt change in the kinetic energy. It happens because the potential energy is changed to $V_{0}$ (due to energy conservation). The new kinetic energy is given by $K_{b}=E_{a}-V_{0}$. The particle travels again the distance b/2 but now going from left to right with constant velocity $v_{b}=\sqrt{2K_{b}/m}$. The time to travel the distance is b/2 is $\Delta t_{b}^{\prime }=b/\left(2v_{b}\right)$.

Now that the particle traveled the distance b/2, it finally reaches the position x=(a+b)/2, which is our Poincaré section. One can conclude that the final energy is $E_{n+1}=E_{a}$ and the final time is $t_{n+1}=t_{n}+\Delta t_{b}+i\Delta t_{a}+\Delta t_{b}^{\prime }$.

We will consider a set of dimensionless variables. It is necessary to reduce the number of variables and control parameters of the system. First, we consider $e=E/V_{0}$, which is the dimensionless energy. ϕ=ωt is the phase of the moving wall. The time to travel some distance is changed to a phase variation by considering: $\Delta \phi_{b}=\omega \Delta t_{b}$, $\Delta \phi_{a}^{\prime }=\omega \Delta t_{a}$ and finally $\Delta \phi_{b}^{\prime }=\omega \Delta t_{b}^{\prime }$. The dimensionless control parameters are r=b/a, which changes the width of the potential well, $\delta =V_{1}/V_{0}$ changes the height of the potential well, and finally $N_{c}=\frac{\omega a}{2\pi }\sqrt{\frac{m}{2V_{0}}}$. $N_{c}$ corresponds to the number of oscillations the moving part completes when a particle travels the distance *a* with energy $E=V_{0}$. It is important to pay attention at 0<δ<1, because if delta has a value greater than 1, we will no longer have the potential well, as the oscillating part may have a potential greater than $v_{o}$. We also can observe that $N_{c}$ is directly proportional to the oscillation frequency of the well ω and inversely proportional to the square root of $V_{0}$. Therefore, increasing the value of $N_{c}$ has the same effect of increasing the angular frequency ω.

Thus, after considering this set of dimensionless variables, we obtain the following map:

(6) $$e_{n+1}=e_{n}+\delta \left[\cos\left(\phi_{n}+\Delta \phi_{b}+i\Delta \phi_{a}\right)-\cos\left(\phi_{n}+\Delta \phi_{b}\right)\right],$$

where *i* is the smallest number that makes the expression $e_{n+1}>1$ true. The final phase is

(7) $$\phi_{n+1}=\left(\phi_{n}+\Delta \phi_{b}+i\Delta \phi_{a}+\Delta \phi_{b}^{\prime }\right)\;\;mod2\pi .$$

The auxiliary variables are given by

(8) $$\Delta \phi_{b}=\frac{\pi N_{c}r}{\sqrt{e_{n}-1}},$$

(9) $$\Delta \phi_{a}=\frac{2\pi N_{c}}{\sqrt{e_{n}-\delta \cos\left(\phi_{n}+\Delta \phi_{b}\right)}},$$

and

(10) $$\Delta \phi_{b}^{\prime }=\frac{\pi N_{c}r}{\sqrt{e_{n+1}-1}}.$$

These dimensionless variables represent, respectively, the time to travel the distance b/2, time to travel distance *a*, and time to travel the distance b/2 before returning to the Poincaré section.

## 3. Results

**Figure 2:**
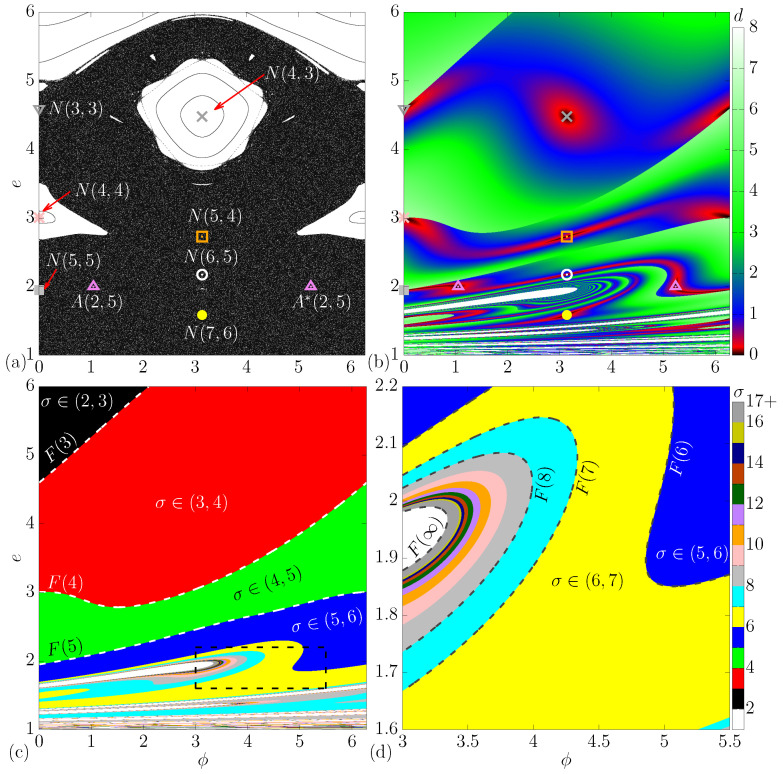
(Color online) Phase space evs.ϕ, where in (**a**), we highlighted the fixed points (elliptic and hyperbolic). In (**b**), the color represents the value of d(e,ϕ), which is given by Equation (17). (**c**,**d**) show colors as the value of σ, which implicitly shows the dispersion of the phase ϕ. (**d**) is an enlargement in the black rectangle shown in (**c**). For all graphs, we considered the following combination of control parameters: $N_{c}=3$, r=1 and δ=0.5.

Figure 2a shows the phase space evs.ϕ. Observe that the phase in such a plot is mod(2π). As one can see, the phase space is of mixed-type, containing a set of periodic islands, a large chaotic sea, and some invariant spanning curves limiting the size of the chaotic sea.

We can obtain the period-1 fixed points by considering $\phi_{n+1}=\phi_{n}=\phi^{*}+2\pi l$ and $e_{n+1}=e_{n}=e^{*}$ in Equations (6) and (7), where *l* is an integer value and $\left(\phi^{*},e^{*}\right)$ is the position of the fixed point. When solving for $e_{n+1}=e_{n}=e^{*}$, we found out the expression

(11) $$\cos\left(\phi^{*}+\Delta \phi_{b}+i\Delta \phi_{a}\right)=\cos\left(\phi^{*}+\Delta \phi_{b}\right).$$

Equation (11) yields: (i) $i\Delta \phi_{a}=2\pi k$ or (ii) $i\Delta \phi_{a}=2\pi k-2\left(\phi^{*}+\Delta \phi_{b}\right)$, where *k* is also an integer number.

Considering case (i), and taking $i\Delta \phi_{a}=2\pi k$ to Equation (7), it is possible to find an analytical result, where the position $\left(\phi^{*},e^{*}\right)$ of the period-1 fixed point can be obtained when

(12) $$e^{*}=1+{\left(\frac{N_{c}r}{l-k}\right)}^{2}.$$

$\phi^{*}$ assumes two possible values

(13) $$\phi^{*}=\arccos\left\{\frac{1}{\delta }\left[e^{*}-{\left(\frac{N_{c}}{k}\right)}^{2}\right]\right\}-\frac{\pi N_{c}r}{\sqrt{e^{*}-1}},$$

or

(14) $$\phi^{*}=2\pi -\arccos\left\{\frac{1}{\delta }\left[e^{*}-{\left(\frac{N_{c}}{k}\right)}^{2}\right]\right\}-\frac{\pi N_{c}r}{\sqrt{e^{*}-1}}.$$

We use the following notation A(k,l) when talking about the period-1 fixed points obtained analytically. As an example, in Figure 2a,b, we show the fixed point A(2,5), so the position $\left(\phi^{*},e^{*}\right)$ is obtained analytically through Equations (12) and (13). $A^{*}\left(k,l\right)$ is the fixed point obtained through Equations (12) and (14), where Figure 2a shows $A^{*}\left(2,5\right)$ as an example. Both A(2,5) and $A^{*}\left(2,5\right)$ are hyperbolic fixed points.

Let us now discuss the case (ii). When we take $i\Delta \phi_{a}=2\pi k-2\left(\phi^{*}+\Delta \phi_{b}\right)$ to Equation (7), we obtain the following expression:

(15) $$e^{*}-\delta \cos\left[\pi \left(k-l\right)+\Delta \phi_{b}\right]-{\left(\frac{\pi N_{c}}{\pi l-\Delta \phi_{b}}\right)}^{2}=0,$$

which needs to be solved numerically to find $e^{*}$. The phase $\phi^{*}$ is then obtained by the following expression

(16) $$\phi^{*}=\pi \left(k-l\right).$$

We use N(k,l) to represent the period-1 fixed points obtained numerically through Equations (15) and (16). Figure 2a shows the result for the numerical fixed points. As one can see, there exists a clear sequence of fixed points arising. For example, on the left of the figure, we have N(3,3), N(4,4) and N(5,5) following a sequence where the values of *k* and *l* are both increased by one unit each. In the middle of the figure, we observe another sequence, where the fixed points N(4,3), N(5,4), N(6,5) and N(7,6) arise. It is nice to observe that N(4,3) and N(4,4) are stable fixed points (elliptic), which are inside periodic islands, while N(3,3), N(5,5), N(5,4), N(6,5), N(7,6), A(2,5) and $A^{*}\left(2,5\right)$ are unstable fixed points (hyperbolic).

### 3.1. Observable d(e,ϕ)

Now, we show an observable named d(e,ϕ). Observe that unstable fixed points, as shown in Figure 2a, cannot be observed when looking directly at the phase space, and we can only highlight them by solving the periodicity condition. This observable d(e,ϕ) can show both stable and unstable fixed points, where we define it as

(17) $$d\left(e,\phi \right)=\sqrt{{\left(\phi_{n+1}-\phi_{n}\right)}^{2}+{\left(e_{n+1}-e_{n}\right)}^{2}},$$

where $\phi_{n+1}$ is mod(2π). The result is plotted in Figure 2b, where in order to construct this figure, we considered a grid of 1000 by 1000 different values for the initial conditions $\phi_{n}\in \left(0,2\pi \right)$ (horizontal axis) and $e_{n}\in \left(1,6\right)$ (vertical axis). So, we iterate the map once and use a color palette that represents the value of d(e,ϕ). One important result found appears when the color tends to black (d→0), where in such cases, we have the position of a fixed point. Thus, it highlights both elliptical and hyperbolic ones. The fixed points found by cases (i) and (ii) confirm that this observation is true, so using d(e,ϕ) is the fastest way to observe fixed points without the necessity of solving equations.

If our aim is to highlight period-2 fixed points; the only necessary change is to consider $\phi_{n+2}$ and $e_{n+2}$ instead of $\phi_{n+1}$ and $e_{n+1}$ in Equation (17). It is unnecessary to go further and study higher-order periodic fixed points because we have another information arising in Figure 2b. As one can see, there exist abrupt changes of color, for example, near the fixed point N(3,3), where the color tends to be red/black, but going a little above, we see that the color tends to be green. There are several curves such as that presenting a complex behavior. Our goal is to understand what happens near them.

### 3.2. Observable σ

We define another observable, named as σ, which measures how the phase ϕ spreads along this axis. We define this observable as

(18) $$\sigma =\frac{\phi_{n+1}}{2\pi },$$

where σ∈ℜ. It is important to emphasize that the function mod is not applied in the phase ϕ, so $\phi_{n+1}$ can assume greater values than 2π. If σ∈(0,1), we can interpret that $\phi_{n+1}\in \left(0,2\pi \right)$. For σ∈(1,2), we know that $\phi_{n+1}\in \left(2\pi ,4\pi \right)$, and so on. We then showed the result in Figure 2c. The way to construct this figure is like the one shown in Figure 2b, but now, the color represents the value of σ. As shown in the color palette presented on the right side of the figure, for σ∈[2,3), we use the black color, while for σ∈[3,4), we use the red color and so on. If σ∈[16,∞), we use the dark-gray color. The white regions have i>1, i.e., these are regions with multiples reflections, which at first moment will be disregarded.

As one can see in Figure 2c, there exists a clear line separating the black (σ∈(2,3)) from the red regions (σ∈(3,4)). To obtain this line, we need to isolate $\phi_{n+1}$ in Equation (18) and bring this to Equation (7). As a result, we find a transcendental equation

(19) $$F\left(\sigma \right)=\phi_{n}-2\pi \sigma +\Delta \phi_{b}+i\Delta \phi_{a}+\Delta \phi_{b}^{\prime },$$

that needs to be solved numerically for $F\left(\sigma \right)=0$ to find the values of $\left(\phi_{n},e_{n}\right)$ that are solutions. The results obtained are plotted as dashed curves in Figure 2c,d. As one can see, curve F(3) is the one that separates the red and black regions. F(4) separates the red [σ∈(3,4)] and green [σ∈(4,5)] regions, and so on, following a logical sequence tending to infinite.

Figure 2d shows an enlargement in the black rectangle of Figure 2c. Here, we show with more details that a sequence of curves F(6),F(7),F(8),⋯,F(∞) is observed. It is possible to show that every time that σ→∞ or F(∞)→∞, we have the boundary that separates regions with and without multiple reflections. As an example, the white-colored regions in Figure 2c,d are regions with multiple reflections (i.e., i>1). We find this border by finding the curve F(∞).

Considering σ→∞ and bringing this to Equation (7), one can conclude that $\Delta \phi_{b}\to \infty$, $\Delta \phi_{a}\to \infty$ or $\Delta \phi_{b}^{\prime }\to \infty$. In the first option, $\Delta \phi_{b}\to \infty$ is meaningless because according to Equation (8), for that, it is necessary that $e_{n}\to 1$ and so the particle would never escape the well. The second option, $\Delta \phi_{a}\to \infty$ given by the Equation (9) is also not viable for the same reason and furthermore, it is mathematically impossible for the denominator of this equation to be zero, since we have $a_{n}>1$ and we have already seen that it is necessary to have 0<δ<1. So, we only have to use the third option, $\Delta \phi_{b}^{\prime }\to \infty$, which is the only one of the three options that makes physical and mathematical sense. As we already know, $\Delta \phi_{b}^{\prime }$ is given by Equation (10), so $\Delta \phi_{b}^{\prime }\to \infty$ has as a possible solution $e_{1}\to 1$. The idea is to solve this equation, which can be done by finding the root of G(i)=0, which is given by

(20) $$G\left(i\right)=e_{0}-1+\delta \left[\cos\left(\phi_{0}+\Delta \phi_{b}+i\Delta \phi_{a}\right)-\cos\left(\phi_{0}+\Delta \phi_{b}\right)\right].$$

An important result appears when we find the solution of G(1)=0, which is the same curve F(∞) in Figure 2d. Therefore, we finally have the border that separates regions containing multiple reflections.

### 3.3. Multiple Reflections

**Figure 3:**
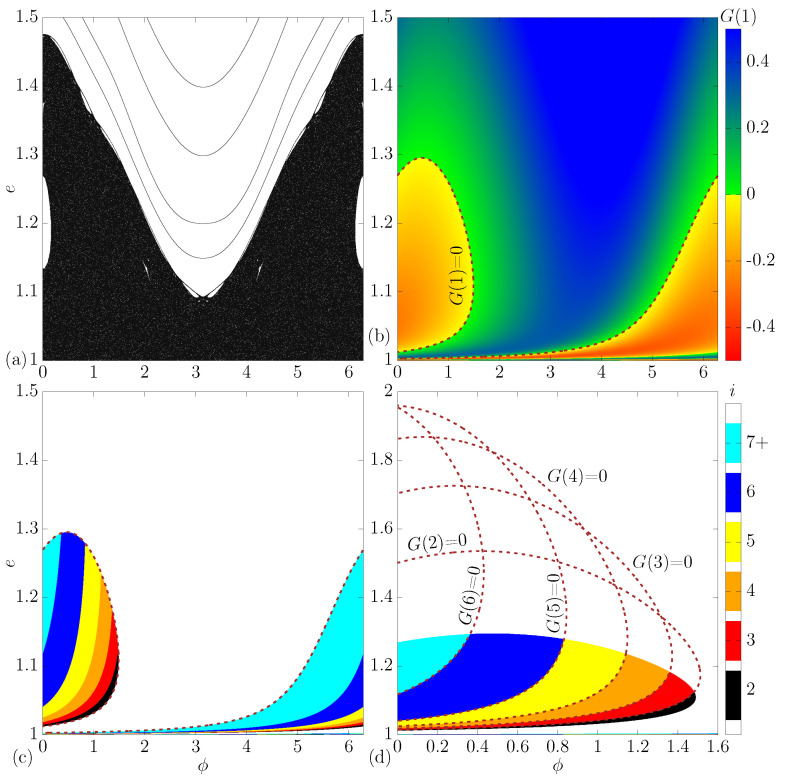
(Color online) In (**a**) phase space evs.ϕ. We used the following set of control parameters: $\left(N_{c};r;\delta \right)=\left(0.1;1;0.5\right)$. In (**b**), we highlight as colors the value of G(1). When G(1)=0, we find the border that separates regions with multiple reflections (i>1) from the region without multiple reflections (i=1). In (**c**,**d**), the colors represent the value of *i* when i>1, e.g., we highlight regions with multiple reflections.

In this section, we show details in the multiple reflections regions. Figure 3a shows the phase space for $N_{c}=0.1$, r=1 and δ=0.5. The phase space shows a large chaotic sea, with some periodic islands and invariant spanning curves.

To highlight the regions containing multiple reflections (i>1), one can start by looking at Figure 3b. In this plot, we considered a grid of 1000 by 1000 different values of $\phi_{0}\in \left(0,2\pi \right)$ and $e_{0}\in \left(1,1.5\right)$, and for each combination of initial conditions, we calculated G(1) through Equation (20). We used a continuous color pallete, from red to yellow (negative values) and green to blue (positive values). Negative values of G(1) characterize regions with multiple reflections (i>1), while positive values mark regions without multiple reflections (i=1). As said before, the border that marks regions with and without multiple reflections is obtained by solving G(1)=0.

Now that we know the location of the regions with multiple reflections (i>1), one can build Figure 3c, where again a grid of 1000 by 1000 different values of $\phi_{0}$ and $e_{0}$ is considered, but the color represents the value of *i*. As one can see, there exists a logical sequence of *i* starting from i=2 (black), i=3 (red) and tending to infinity in the cyan region (i≥7).

Figure 3d shows an enlargement of Figure 3c. Here, we show more details about how to obtain the border that separates regions with different values of *i*. The border that separates i=2 from i=3 is obtained by solving G(2)=0 (see Equation (20)). The other separations were also obtained by solving G(3)=0, G(4)=0, G(5)=0, G(6)=0 and so on.

**Figure 4:**
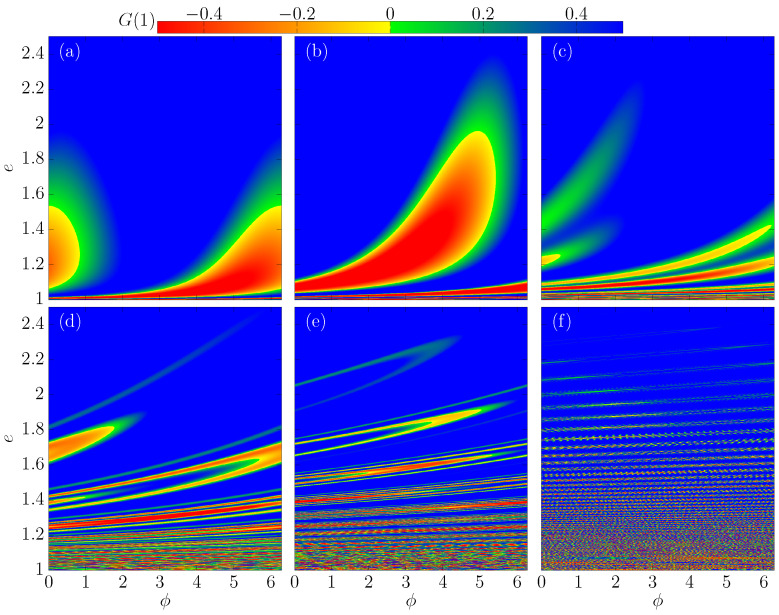
(Color online) Phase space evs.ϕ for different values of $N_{c}$. We use (**a**) $N_{c}=0.2$, (**b**) $N_{c}=0.5$, (**c**) $N_{c}=1$, (**d**) $N_{c}=5$, (**e**) $N_{c}=10$ and (**f**) $N_{c}=50$. In all simulations, the following set of control parameters was used $\left(r;\delta \right)=\left(1;0.5\right)$.

As a comparison, Figure 4a–f shows $e_{0}\;\mathrm{vs}.\;\phi_{0}$, and the color is the value of G(1). Our goal is to highlight regions with multiple reflections (i>1) for different values of $N_{c}$. The value of $N_{c}$ is equal to (a) 0.2, (b) 0.5, (c) 1, (d) 5, (e) 10 and (f) 50. We kept constant the value of the other control parameters by setting r=1 and δ=0.5. As one can see, if we increase the value of $N_{c}$, it will considerably change the complexity of the curves. We can see that regions with multiple reflections are stretching along the phase space, but for all situations, the regions with i>1 are limited to energies below 2.4. Figure 2a shows us that the chaotic sea can be observed for e≲6, and when we increase the value of $N_{c}$, this energy grows quick.

### 3.4. Histogram for Multiple Reflections

Here, we will study the area occupied by the regions with i>1. To do this, we started by considering the following simulation: we take a grid containing *M* by *M* different values of $\phi_{0}\in \left(0,2\pi \right)$ and $e_{0}\in \left(1,2.5\right)$, and for each combination of $\left(\phi_{0},e_{0}\right)$, we check the value of *i* after one iteration. After that, we accumulate it in a histogram *H*. So, the histogram counts the number of initial conditions that started in a i>1 region. It is an indirect way to measure the area occupied by *i*, i.e., the greater the value of *H*, the greater the area.

**Figure 5:**
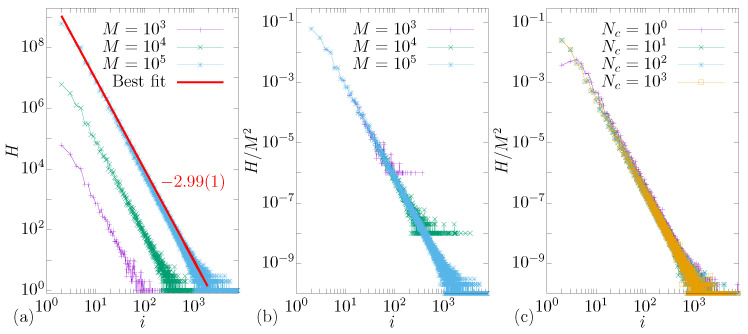
(Color online) Histogram *H* as function of *i* for different values of *M* in (**a**). After a suitable rescale in the vertical axis $\left(H\to H/M^{2}\right)$, all curves present a universal behavior, showing a scaling invariance in (**b**). In (**c**), we show that changing the value of $N_{c}$ does not affect the position of the curves. For these simulations, we fixed the other control paramters, i.e., r=1 and δ=0.5.

Figure 5a shows the result obtained when considering different values grid-size *M*. As one can see, when increasing *M*, the curves of *H* go to the right. If we apply a re-scale in the vertical axis, i.e., $H\to H/M^{2}$, we see that all curves have a universal behavior, with a slope (for a power-law fit) near −3. With this result, we prove a scaling invariance of the plot of Hvs.i; i.e., we can take any value of *M*, and the behavior is described by the same curve.

Now, we considered $M=10^{5}$, and we changed the value of $N_{c}$ considering it equal to $10^{0}$, $10^{1}$, $10^{2}$, and $10^{3}$. As one can see, independently of the value of $N_{c}$, all curves stay at the same position, so, as we said before, we can argue that the area of the regions with multiple reflections is constant for any $N_{c}$ taken. These regions *i* stretch and shrink in several directions, as shown in Figure 4a–f, but the area seems to be constant.

### 3.5. Density Plots in the $e_{0}e_{1}$ Plane

In the last part of the paper, we show some interesting structures that appear in the $e_{0}e_{1}$ plane. Again, a grid of *M* by *M* different values of $\phi_{0}\in \left(2,\pi \right)$ and $e_{0}\in \left(1,5\right)$ is taken, and for each combination of $\left(\phi_{0},e_{0}\right)$, we find the corresponding value of $e_{1}$. Observe that $M=10^{4}$ for all next results. Now, we can plot $e_{1}\;\mathrm{vs}.\;e_{0}$ directly, but this result does not reveal much information. It is better to consider a density plot, which is completed as follows: we count the number of trajectories (points) that visited boxes in a grid of 1000×1000 equally spaced intervals in the $e_{1}e_{0}$ plane. We define the quantity ξ as the logarithm of the number of trajectories that visit a given box Σ:

(21) $$\xi =\log\left(\Sigma \right).$$

**Figure 6:**
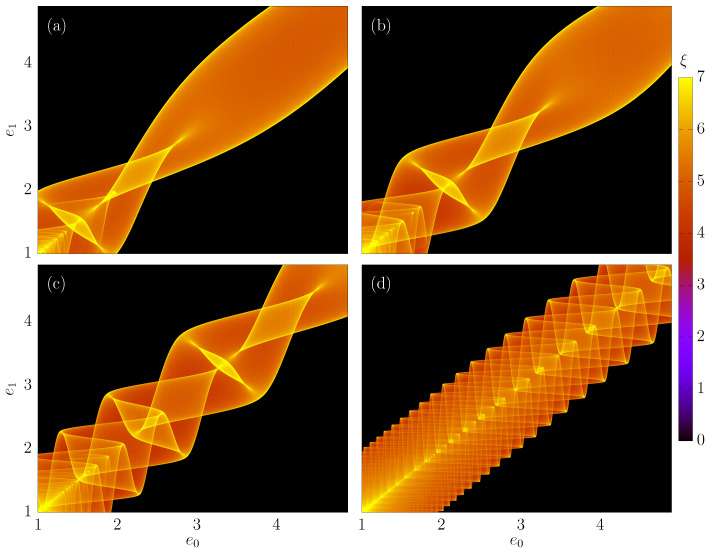
(Color online) Density plots $e_{0}e_{1}$ where the colors show the value of ξ given by Equation (21). In (**a**–**d**), different values of $N_{c}$ are taken. We used (**a**) $N_{c}=3$, (**b**) $N_{c}=5$, (**c**) $N_{c}=10$ and (**d**) $N_{c}=50$. The other control parameters were fixed as r=1 and δ=0.5.

The log function is used to suppress regions with huge counts. Figure 6a–d show us the results for different values of $N_{c}$ and constant values of (r,δ)=(1,0.5). In (a), we considered $N_{c}=3$, in (b) $N_{c}=5$, in (c) $N_{c}=10$ and in (d) $N_{c}=50$. We see a very complicated behavior, and more details need to be extracted.

**Figure 7:**
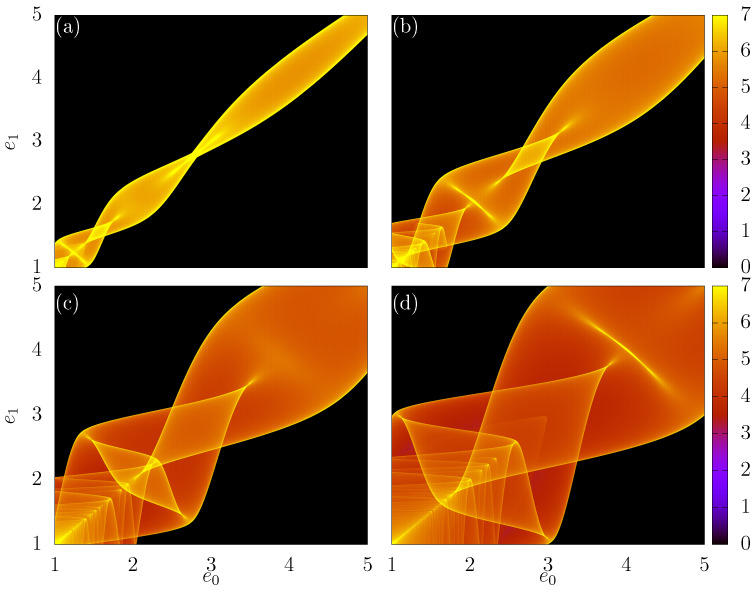
Density plots $e_{0}e_{1}$ where the colors show the value of ξ given by Equation (21). In (**a**–**d**), different values of δ are taken. We used (**a**) δ=0.2, (**b**) δ=0.4, (**c**) δ=0.7 and (**d**) δ=1. The other control parameters were fixed as r=1 and $N_{c}=0.5$.

**Figure 8:**
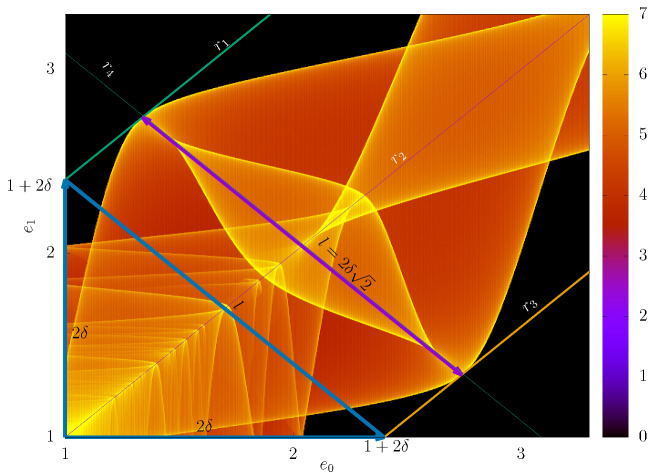
Density plots $e_{0}e_{1}$ where the colors show the value of ξ given by Equation (21). We used r=1, $N_{c}=0.5$ and δ=0.7.

In our simulations, we noticed that when we changed the value of the control parameter δ, the width of the structure found in the plane $e_{0}e_{1}$ changed depending on this parameter; that is, when we increased or decreased the value of δ, the width of the structure also increased or decreased, as we can see in Figure 7, where we plot this structure for δ=0.2, δ=0.4, δ=0.7 and δ=1, keeping the values of $N_{c}=5$ and r=1 constant. We then decided to investigate the existence of a mathematical relationship between δ and the width of such a structure. To do this, first, we draw the line $e_{1}=e_{0}$, through which we can see that the structure is symmetric with respect to this line. Next, we draw parallel lines with the line $e_{1}=e_{0}$, tangent to the structures at their outermost points, and carefully analyze the values where the parallel lines cut the coordinate axes of the plane $e_{0}e_{1}$. We note that this point is always given by 1+2δ, which means that the distance from the point $\left(e_{0},e_{1}\right)=\left(1,1\right)$ to the intersection point of lines parallel to $e_{1}=e_{0}$ is always 2δ on both axes, as shown in Figure 8, which is an enlargement of Figure 7c, where we call the line $e_{1}=e_{0}$ from $r_{2}$ and from $r_{1}$ and $r_{3}$ the lines parallel to $r_{2}$ that are tangent the structures. In this figure, we draw a line segment of size *l*, joining the intersection points of the parallel lines with $e_{1}=e_{0}$ with the coordinate axes. Note that the length of *l* will have exactly the same measurement as the maximum width of the structure, as shown by the straight line under the line $r_{4}$. Thus, in the isosceles right triangle that was formed, we can apply the Pythagorean Theorem and conclude that

(22) $$l=2\delta \sqrt{2}.$$

The mathematical explanation for this relationship is given by the Equation (6). We can note that in this equation, the greatest value that $\cos\left(\phi_{n}+\Delta \phi_{b}+i\Delta \phi_{a}\right)$ mode assumes is 1 and the greatest value that $-\cos\left(\phi_{n}+\Delta \phi_{b}\right)$ can assume that it is also 1. When this happens, we have that $\Delta e_{max}$, which is the greatest distance between two consecutive values of energy is given by

(23) $$\Delta e_{max}=e_{n+1}-e_{n}=2\delta ,$$

where $\Delta e_{max}$, which is exactly the distance from the point (1,1) to the points where the lines tangent to the structures cut the axes $e_{0}$ and $e_{1}$ of the plane $e_{0}e_{1}$.

**Figure 9:**
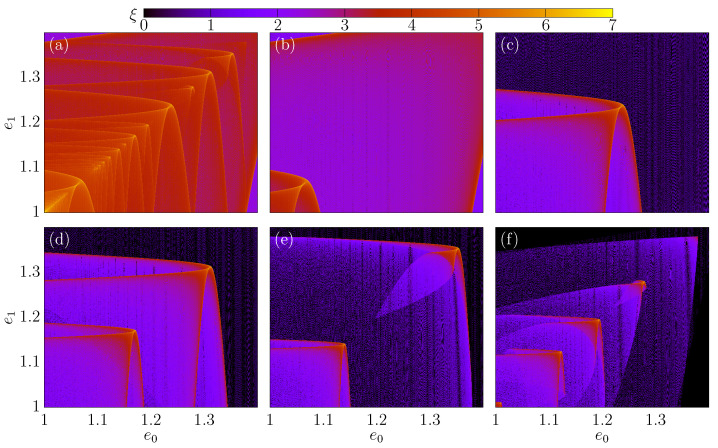
(Color online) Density plots, where in the horizontal and vertical axis, we have $e_{0}\in \left(1,1.4\right)$ and $e_{1}\in \left(1,1.4\right)$, respectively. In (**a**), we show the complete scenario, where all values of *i* are shown. Item (**b**) highlights only structures with i=1, in (**c**) i=2, in (**d**) i=3, in (**e**) i=4 and finally in (**f**) i=5.

Let us focus on Figure 6a. We will consider an enlargement in the region $e_{0}\in \left(1,1.4\right)$ and $e_{1}\in \left(1,1.4\right)$, where the result is plotted in Figure 9a. We see several structures, which, apparently, does not present a pattern. It differs from the results obtained for a time-dependent potential barrier in a previous work [25]. In that work, it was possible to show the structures had an auto-similar pattern, but here, the structures are more complex. Basically, the structures shown in Figure 9a are structures with different values of *i*. For example, Figure 9b shows only structures with i=1 (without multiple reflections).

Figure 9c shows the structures with i=2. As one can see, a single structure appeared. Figure 9d shows the structures with i=3, where two structures appear. It is interesting to observe Figure 9e which presents i=4. The second structure observed in the right portion of the plot seems to be incomplete. These incomplete structures can also be seen in Figure 9f, which highlights only one structure with i=5. The five structures observed are incomplete.

When increasing the value of *i*, we will check an increased number of structures, which contribute to the last scenario shown in Figure 9a. It is important to mention that similar structures appear, for example, in the Fermi–Ulam model [28], in concave billiards [29,30] and in the time-dependent potential barrier [25], but more studies are necessary to understand completely the mechanism that produces these structures.

## 4. Conclusions

Summarizing, we studied the dynamics of a classical particle confined to move in a time-dependent potential well. The bottom of the well is allowed to oscillate in time. Three parameters control the dynamics of the particle, which is made via a two-dimensional, nonlinear and area preserving mapping written in terms of energy and phase. Since there is no gradient of the potential, a particle moves with a constant velocity inside of the potential changing the velocity only when it departs from region *a* to region *b* or vice versa. The phase space, which gives the specification of all possible states for the dynamics, is of mixed form exhibiting then large chaotic seas, islands of periodicity, and a set of invariant spanning curves, limiting the chaotic diffusion. The expression of the period one fixed points are obtained analytically, while larger periods are obtained numerically. The multiple reflections, which denote a rare event for the dynamics along the phase space, are investigated from the density of points σ inside of the reflection zone. It is shown in a color plate with a degrade of colors showing differences of intensity, hence proving different concentrations. In fairness, there is a set of continuous curves separating regions where multiple reflections are observed in energy vs. energy plane.
